# Treatment with the Glycosphingolipid Modulator THI Rescues Myelin Integrity in the Striatum of R6/2 HD Mice

**DOI:** 10.3390/ijms24065956

**Published:** 2023-03-22

**Authors:** Giuseppe Pepe, Paola Lenzi, Luca Capocci, Federico Marracino, Ludovica Pizzati, Pamela Scarselli, Alba Di Pardo, Francesco Fornai, Vittorio Maglione

**Affiliations:** 1 IRCCS Neuromed, Via Dell’elettronica, 86077 Pozzilli, Italy; 2 Department of Translational Research and New Technologies in Medicine and Surgery, University of Pisa, Via Roma 55, 56126 Pisa, Italy

**Keywords:** HD, myelin, neurodegeneration, glycosphingolipids

## Abstract

Huntington’s disease is one of the most common dominantly inherited neurodegenerative disorders caused by an expansion of a polyglutamine (polyQ) stretch in the N-terminal region of huntingtin (Htt). Among all the molecular mechanisms, affected by the mutation, emerging evidence proposes glycosphingolipid dysfunction as one of the major determinants. High levels of sphingolipids have been found to localize in the myelin sheaths of oligodendrocytes, where they play an important role in myelination stability and functions. In this study, we investigated any potential existing link between sphingolipid modulation and myelin structure by performing both ultrastructural and biochemical analyses. Our findings demonstrated that the treatment with the glycosphingolipid modulator THI preserved myelin thickness and the overall structure and reduced both area and diameter of pathologically giant axons in the striatum of HD mice. These ultrastructural findings were associated with restoration of different myelin marker protein, such as myelin-associated glycoprotein (MAG), myelin basic protein (MBP) and 2′, 3′ Cyclic Nucleotide 3′-Phosphodiesterase (CNP). Interestingly, the compound modulated the expression of glycosphingolipid biosynthetic enzymes and increased levels of GM1, whose elevation has been extensively reported to be associated with reduced toxicity of mutant Htt in different HD pre-clinical models. Our study further supports the evidence that the metabolism of glycosphingolipids may represent an effective therapeutic target for the disease.

## 1. Introduction

Huntington’s disease (HD), the most common dominantly inherited neurodegenerative disorder, is characterized by the progressive striatal and cortical neurodegeneration and associated motor, cognitive and behavioral disturbances. The disease-causing mutation is the expansion of a polyglutamine (polyQ) stretch in the N-terminal region of huntingtin (Htt), a ubiquitous protein with multiple functions [1,2]. Expansion of the polyQ stretch endows mutant Htt (mHtt) with toxic properties and results in a broad array of cell dysfunctions [3].

As consequence of the polyQ expansion, mutant protein changes its folding resulting in the formation of soluble oligomers that are prone to form aggregates [1]. Formation of intranuclear inclusions of mHtt is a pathological hallmark of the disease that may conceivably cause neuronal dysfunction and neuronal degeneration [1].

Although neurons are reported to be primarily affected, other cell populations may be susceptible to the disease [4]. mHtt exerts adverse effects also in glial cells by interfering with the functional intracellular pathways [5]. Animal studies describe white matter segmentation and myelin breakdown in HD mouse models at different stages of the disease [6]. Although the mechanism by which mHtt negatively affects myelin integrity is not completely understood, recently it has been reported that expression of mHtt in oligodendrocytes leads to reduction in myelin basic protein (MBP), the major constituent of myelin sheaths [7]. This evidence recapitulates the loss of brain white matter, recently described in either symptomatic or pre-symptomatic subjects [6], and suggests that white matter dysfunctions may be important in HD pathogenesis and may be likely identified as an early event of this devastating disorder.

Among all the dysfunctional molecular mechanisms that have been reported to play a critical role in the pathogenesis of HD, emerging evidence proposes lipid dysfunction as one critical determinant [8,9,10,11,12] and, in particular, the defective metabolism of (glyco)sphingolipids may represent a possible promising therapeutic target [13,14,15,16,17,18].

High levels of sphingolipids have been found to localize in the myelin sheaths of oligodendrocytes where they play a fundamental role in the initiation of myelination as well as in the maintenance of the myelin integrity and in the regulation of axon–glia interactions [19]. Mice lacking specific glycosphingolipids, such as gangliosides GM1 and GD1a in the central nervous system (CNS), show oligodentrocyte defects and reduced stability of myelin paranodal junctions [20,21]. Mice knockout for ganglioside-synthetizing enzymes are characterized by axonal degeneration, increased unmyelinated fibers with disruption of axonal-glia interaction, and perturbed myelin paranodal stability [21]. These alterations may precede motor dysfunctions by several months and recapitulate the early white matter dysfunctions described in pre-symptomatic HD patients [22] as well as myelin degeneration observed in R6/2 HD mice [23,24]. Modulation of (glyco)sphingolipid pathways is beneficial in HD mice and it is associated with increased levels of myelin proteins in the corpus callosum [18,25]. Whether this approach may be beneficial also at striatal level has not been investigated.

Here, we assessed any possible existing link between the modulation of sphingolipids by THI [18] and myelin structure in the striatum of R6/2 mice by performing both ultrastructural and biochemical analyses.

Our findings demonstrated that the treatment with THI preserved myelin thickness and the overall structure while reducing pathologically the enlarged axon area and the diameter in HD mice.

The ultrastructural findings were associated with normal expression of different myelin marker protein such as myelin-associated glycoprotein (MAG), myelin basic protein (MBP), and 2′, 3′ Cyclic Nucleotide 3′-Phosphodiesterase (CNP). Interestingly, the compound modulated the expression of ganglioside biosynthetic enzymes and increased GM1 levels.

## 2. Results

### 2.1. THI Rescues Axonal Myelination Loss within Striatum of R6/2 Transgenic Mice

A number of evidence indicates that both human patients and HD animal models are characterized by myelination deficits [26,27,28].

We have recently demonstrated that the inhibition of SGPL1 by THI restored myelin organization in the corpus callosum of R6/2 mice [18]. In order to verify whether the compound might exert similar effect in the striatum, we performed ultrastructural analyses in HD and control mice.

**Figure 1:**
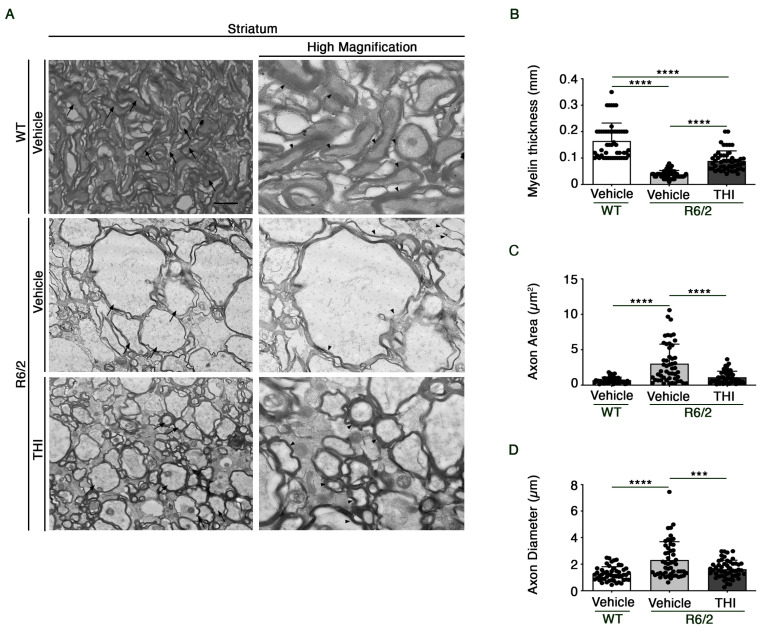
THI rescues axonal myelination loss within striatum from R6/2 transgenic mice. Representative electron micrographs of axons (**A**) in the striatum from vehicle-treated WT and vehicle- and THI-treated R6/2 mice at 11 weeks of age. Giant axons (left panels) are indicated by arrows. Myelin sheet abnormalities (right panels) are indicated by arrowheads. Scale bar (left panels): 1 µm; Scale bar (right panels; high magnification): 0.2 µm. Bar graphs representing the myelin thickness (**B**), the axon area (**C**) and the axon diameter (**D**) in the striatum from vehicle-treated WT and vehicle- and THI-treated R6/2 mice at 11 weeks of age. Values are represented as mean ± SD. *n* = 4 for each group of mice. Fifty axons for each experimental group. One-way ANOVA, *** *p* < 0.001; **** *p* < 0.0001.

Electron microscopy analysis revealed an aberrant white matter structure with a dramatic loss of myelin sheaths and an increased axon dimension in the striatum of R6/2 mice compared with WT littermates (Figure 1A). Interestingly, administration of THI preserved myelin thickness (One-way ANOVA, F = 98.53, *p* < 0.0001) (Figure 1B) and normalized both the axon area (One-way ANOVA, F = 27.73, *p* < 0.0001) and the diameter (One-way ANOVA, F = 16.24, *p* < 0.0001) in HD mice (Figure 1C,D).

### 2.2. THI Treatment Preserves the Expression of Myelin-Related Proteins and Genes in the Striatum of R6/2 Mice

To further investigate whether amelioration of myelin structure was paralleled by changes in the expression of myelin components, we assessed the expression profiles of MBP (myelin basic protein), MAG (myelin-associated glycoprotein), CNP (2′, 3′ Cyclic Nucleotide 3′-Phosphodiesterase), MOG (myelin oligodendrocyte glycoprotein), and PLP (proteo-lipid protein), a specific marker of myelin. 

**Figure 2:**
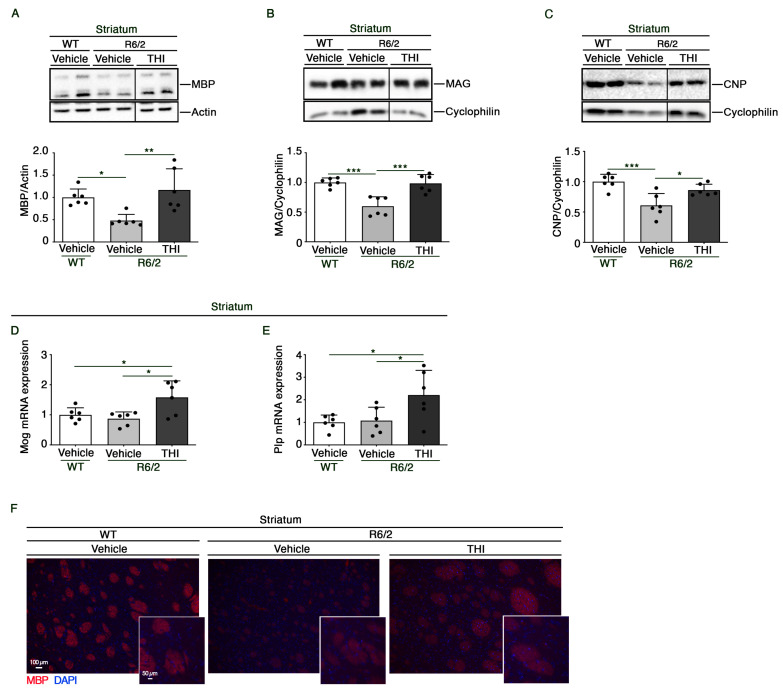
THI treatment preserves the expression of myelin-related proteins and genes in the striatum of R6/2 mice. Representative cropped immunoblottings and densitometric analysis of MBP (**A**), MAG (**B**) and CNP (**C**) in striatal tissues from vehicle-treated WT and vehicle- and THI-treated R6/2 mice at 11 weeks of age. In each immunoblotting, all samples were run on the same gel. Non-adjacent samples are separated by a black line. Quantitative PCR analysis of Mog (**D**) and Plp (**E**) in striatal tissues from vehicle-treated WT and vehicle- and THI-treated R6/2 mice at 11 weeks of age. Representative fluorescence microscopy micrograph of MBP (**F**) (red signal) in the striatum of vehicle-treated WT and vehicle- and THI-treated R6/2 mice at 11 weeks of age. Nuclei are stained with DAPI (blue signal). Scale bars: 100 μm and 50 μm. Values are represented as mean  ±  SD. *n* = 6 for each group of mice. One-Way ANOVA, * *p* < 0.05; ** *p* < 0.01; *** *p* < 0.001.

Treatment with THI preserved normal protein levels of MBP (One-way ANOVA, F = 8.232, *p* = 0.0039) (Figure 2A,F), MAG (One-way ANOVA, F = 17.48, *p* = 0.0001) (Figure 2B) and CNP (One-way ANOVA, F = 11.42, *p* = 0.0010) (Figure 2C) and increased mRNA levels of Mog (One-way ANOVA, F = 6.365, *p* = 0.0100) (Figure 2D) and Plp (One-way ANOVA, F = 5.002, *p* = 0.0217) (Figure 2E), as assessed by immunoblotting and qPCR analyses, respectively.

### 2.3. Administration of THI Modulates Metabolism of Gangliosides in the Striatum of R6/2 Mice

Previous studies indicated that the metabolism of gangliosides is aberrant in different HD settings and that administration of exogenous GM1 exerts beneficial effects in different HD in vivo models [9,10,13,14].

Given the pivotal role that gangliosides play in myelin stability and functions, we investigated whether the beneficial effect of THI may be eventually associated with the modulation of ganglioside metabolism.

**Figure 3:**
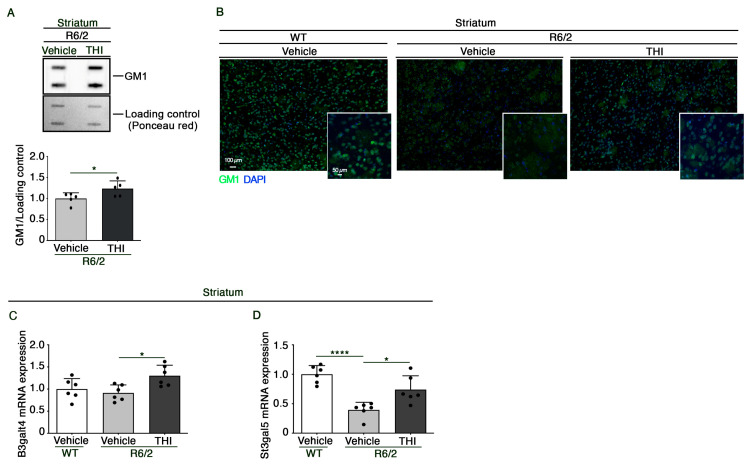
Administration of THI modulates ganglioside metabolism in the striatum of R6/2 mice. Representative cropped immunoblottings and densitometric analysis of GM1 (**A**) in striatal tissues from vehicle- and THI-treated R6/2 mice at 11 weeks of age. Representative fluorescence microscopy micrograph of GM1 (**B**) (green signal) in the striatum of vehicle-treated WT and vehicle- and THI-treated R6/2 mice at 11 weeks of age. Nuclei are stained with DAPI (blue signal). Scale bars: 100 μm and 50 μm. Quantitative PCR analysis of B3galt4 (**C**) and St3gal5 (**D**) in striatal tissues from vehicle-treated WT and vehicle- and THI-treated R6/2 mice at 11 weeks of age. Values are represented as mean  ±  SD. *n* = 5/6 for each group of mice. Unpaired *t*-test (**A**), One-Way ANOVA, * *p* < 0.05; **** *p* < 0.0001.

Semiquantitative analysis of GM1 demonstrated that the compound was able to increase the content of ganglioside in the striatum of HD mice (Unpaired *t*-test, *t* = 2.317, *p* = 0.0491) (Figure 3A,B). This was associated with increased mRNA levels of GM1 synthase (B3galt4) (One-way ANOVA, F = 5.190, *p* = 0.0194) (Figure 3C) and with the normalization of the expression of GM3 synthase (St3gal5) (One-way ANOVA, F = 17.80, *p* = 0.0001) (Figure 3D), the rate limiting-enzyme of the ganglioside biosynthetic pathway, as assessed by qPCR analysis.

## 3. Discussion

Evidence indicates that both in HD animal models and HD patients have a strong collapse of myelin sheaths that occurs early in the disease course [29]. This alteration is paralleled by the reduced expression of myelin-related genes such as MBP and/or MAG [6].

Early myelin degradation affects nerve impulses and results in defective axonal transport, synaptic loss, and axonal degeneration [30]. Interestingly, evidence indicates that mHtt downregulates myelin-associated gene expression and affects myelin functions [7,31,32].

Recently, we have demonstrated that the administration of THI, a specific inhibitor of S1P lyase (SGPL-1), results therapeutically effective in R6/2 mice by evoking neuroprotective pathways, reducing mHtt aggregates and preserving white matter integrity in the corpus callosum [18].

In this study, we aimed at assessing any possible beneficial effect of THI administration on myelin structure in the striatum of R6/2 mice.

WT mice displayed well-structured myelinated fibers, with typical myelin sheaths surrounding the axons. In contrast, in R6/2 mice we observed giant axons with myelin sheaths lacking typical layers. Increased axon diameter is reported in other neurological diseases [33,34]. We can speculate that such an increase may likely serve as a compensatory mechanism for counteracting fiber demyelination and preserving the normal nerve impulses.

As we previously found, in the corpus callosum of R6/2 mice [18], THI preserved normal myelin structure. Indeed, the treatment restored normal levels of several myelin markers, such as MBP, MAG, and CNP, whose reduction has been reported in different HD settings [6,25], and increased levels of Mog and Plp mRNA expression.

Much evidence proposes that neuronal degeneration can be associated with mHtt-dependent dysfunction in non-neuronal cell types [29,35,36].

Whether myelin changes, reported in this study, were secondary to the neuronal dysfunction or not, is not known. However, the beneficial effect that THI administration exerted on myelin organization suggests a potential involvement of myelin itself in the neuroprotective response previously observed in the striatum of R6/2 mice [18].

Glycosphingolipids are lipids highly represented in the brain, and gangliosides are very abundant in myelin sheaths [21,37]. An aberrant ganglioside metabolism is associated with different neurological disorders, characterized by myelin derangement and axon instability [21].

A number of studies indicate that metabolism of (glyco)sphingolipid is defective in HD [9,10,11,12,38]. Coherently, any intervention aimed at modulating their levels is beneficial and is associated with stimulation of neuroprotective pathways in the striatum of different HD animal models [13,14,15,17,25,39].

We have previously demonstrated that THI restored normal level of Glucosylceramide (GluCer) [18], a pivotal molecule in the synthesis of glycosphingolipids, whose levels have been reported to be increased in HD [18,40,41].

In this study, we report that the compound is also able to increase levels of ganglioside GM1, whose content is usually reduced in the striatum of HD mouse models [9,10].

Altogether these findings suggest that THI may likely restore normal balance among different glycosphingolipid species. This is conceivably due to its ability to stimulate the endomembrane system [18], which is normally involved in the synthesis and recycling of glycolipids.

In conclusion, our findings further support the idea that the defective glycosphingolipid metabolism in HD is druggable. Molecules targeting this pathway may represent a new approach for the treatment of the disease by acting on different types of brain cells.

## 4. Materials and Methods

### 4.1. Animal Model

Breeding pairs of the R6/2 line of transgenic mice [strain name: B6CBA-tgN (HDexon1) 62Gpb/1J] with 160 (CAG) repeat expansions were purchased from Jackson Laboratories. All experimental procedures were approved by the IRCCS Neuromed Animal Care Review Board and by “Istituto Superiore di Sanità” (ISS permit number: 760/2020-PR) and were conducted according to the 2010/63/EU Directive for animal experiments.

### 4.2. In Vivo Drug Administration

THI (Cayman, Ann Arbor, MI, USA, cat. no. 13222) was dissolved in DMSO, further diluted in saline (vehicle), and daily administered by intraperitoneal (i.p.) injection at a dose of 0.1 mg/kg. Control mice were injected daily with the same volume of vehicle containing DMSO [18].

### 4.3. Transmission Electron Microscopy (TEM)

Mouse brains were immersed in the fixing solution (paraformaldehyde 2.0% and glutaraldehyde 1% in 0.1 M phosphate buffer pH 7.4) overnight at 4 °C. After washing in PBS (0.1 M), small blocks from striatum were dissected out and post-fixed in 1% osmium tetroxide (OsO4) for 1 h at 4 °C. After washing in PBS, the samples were dehydrated in a gradient of ethanol solutions and finally embedded in epon-araldite resin. Ultrathin sections (90 nm) were cut at ultra-microtome (Leica Microsystems, Wetzlar, Germany) to be collected on cupper grids and stained with uranyl acetate and lead citrate. Ultrathin sections were observed at electron microscopy (Jeol JEM SX100, Jeol, Tokyo, Japan) at an acceleration voltage of 80 kV. For ultrastructural morphometry, grids containing non-serial ultrathin sections (90 nm thick) were examined at a magnification of 6000×. Several grids were analyzed in order to count a total of 50 axons from the striatum from each experimental group. For the ultrastructural analysis of axons, grids containing non-serial ultrathin sections (90 nm thick) were examined at a magnification of 6000×. Axon maximum diameter; axon area and myelin thickness were assessed.

### 4.4. Brain Lysate Preparation and Immunoblottings

Mice were sacrificed within 1 h after the last treatment by cervical dislocation and brains were immediately snap frozen in liquid N2 and pulverized in a mortar with a pestle, as previously described [18].

For the immunoblottings, proteins (20 μg) were resolved on 10% SDS-PAGE and immunoblotted with the following antibodies: anti-MBP (1:1000) (Cell Signaling, Danvers, MA, USA, cat. no. 13344), anti-MAG (1:1000) (Santa Cruz, cat. no. sc-166849) and anti-CNP (1:1000) (Santa Cruz, CA, USA, cat. no. sc-166558). For protein normalization, anti-actin (1:5000) (Sigma-Aldrich, cat. no. A5441) and anti-cyclophilin (1:2000) (Abcam, Cambridge, UK, cat. no. ab16045) were used. Immunoblots were then exposed to specific HRP-conjugated secondary antibodies (Millipore, Burlington, MA, USA, cat. nos. 401315 and 401215). Protein bands were visualized by ECL and quantified by Image Lab Software Version 6.0 (Bio-Rad Laboratories, Hercules, CA, USA).

### 4.5. Semiquantitative Analysis of Ganglioside GM1

Five micrograms of total protein lysate were spotted on nitrocellulose membrane using a slot-blotting apparatus (Hoefer PR 648 Slot Blot Blotting Manifold). Membranes were blocked in 5% milk in TBS-T and incubated with Cholera Toxin Subunit B horseradish peroxidase conjugate (1:2000) (Millipore, cat. no. C34780). Protein bands were visualized by ECL and quantified by Image Lab Software Version 6.0 (Bio-Rad Laboratories). Ponceau Red staining served as a total protein loading control [18].

### 4.6. Quantitative Real-Time PCR

Total RNA was extracted as previously described [18] and qPCR analysis was performed on a CFX Connect Real-Time System instrument (Bio-Rad Laboratories). The following primers were used (5′→3′): Mog-Fw: AGCTGCTTCCTCTCCCTTCTC; Mog-Rv: ACTAAAGCCCGGATGGGATAC; Plp-Fw: GACATGAAGCTCTCACTGGTAC; Plp-Rv: CATACATTCTGGCATCAGCGC; St3gal5-Fw: AGTCCCACTCCAGCCAAAG; St3gal5-Rv: CCAAGACAACGGCAATGAC; B3galt4-Fw: GGCAGTGCCCCTTCTGTATTT; B3galt4-Rv: CGAGGCATAGGGTGGAAAAG; Cyc-Fw: TCCAAAGACAGCAGAAAACTTTCG; Cyc-Rv: TCTTCTTGCTGGTCTTGCCATTCC.

### 4.7. Histochemical Analyses

Brains were processed, embedded in paraffin wax, and 10 μm coronal sections were cut using a microtome. For the assessment of myelination, brain sections were incubated with anti-MBP antibody (1:100) (Cell Signaling, cat. no. 13344). A goat anti-mouse CY3-conjugate (Millipore, Cat. N. AP124C) was used as secondary antibody. For the assessment of ganglioside GM1, brain sections were incubated with Cholera Toxin Subunit B conjugates with Alexa Fluor 488 (1:100) (Millipore, cat. no. C-34775).

### 4.8. Statistics

An unpaired *t*-test was used in GM1 immunoblotting experiments. One-way ANOVA followed by Tukey post-test was used in immunoblotting and qPCR experiments.

## Data Availability

Data are available from the corresponding author on request.
